# Bistability and hysteresis of the 'Secteur' differentiation are controlled by a two-gene locus in *Nectria haematococca*

**DOI:** 10.1186/1741-7007-2-18

**Published:** 2004-08-16

**Authors:** Stéphane Graziani, Philippe Silar, Marie-Josée Daboussi

**Affiliations:** 1 Institut de Génétique et Microbiologie, UMR CNRS 8621, Bât 400 Université Paris-Sud, 15 rue George Clemenceau, 91405 Orsay Cedex, France

## Abstract

**Background:**

Bistability and hysteresis are increasingly recognized as major properties of regulatory networks governing numerous biological phenomena, such as differentiation and cell cycle progression. The full scope of the underlying molecular mechanisms leading to bistability and hysteresis remains elusive. *Nectria haemaotcocca*, a saprophytic or pathogenic fungus with sexual reproduction, exhibits a bistable morphological modification characterized by a reduced growth rate and an intense pigmentation. Bistability is triggered by the presence or absence of σ, a cytoplasmic determinant. This determinant spreads in an infectious manner in the hyphae of the growing margin, insuring hysteresis of the differentiation.

**Results:**

Seven mutants specifically affected in the generation of σ were selected through two different screening strategies. The *s*_1 _and *s*_2 _mutations completely abolish the generation of σ and of its morphological expression, the Secteur. The remaining five mutations promote its constitutive generation, which determines an intense pigmentation but not growth alteration. The seven mutations map at the same locus, *Ses *(for 'Secteur-specific'). The *s*_2 _mutant was obtained by an insertional mutagenesis strategy, which permitted the cloning of the *Ses *locus. Sequence and transcription analysis reveals that *Ses *is composed of two closely linked genes, *SesA*, mutated in the *s*_1 _and *s*_2 _mutant strains, and *SesB*, mutated in the s* mutant strains. *SesB *shares sequence similarity with animal and fungal putative proteins, with potential esterase/lipase/thioesterase activity, whereas *SesA *is similar to proteins of unknown function present only in the filamentous fungi *Fusarium graminearum *and *Podospora anserina*.

**Conclusions:**

The cloning of *Ses *provides evidence that a system encoded by two linked genes directs a bistable and hysteretic switch in a eukaryote. Atypical regulatory relations between the two proteins may account for the hysteresis of Secteur differentiation.

## Background

Although it has long been known that cellular memory, or epigenetic states, can be created by emergent properties of regulatory or metabolic networks (see Delbrück in the discussion of [1], and [2,3]), the full implications of this type of inheritance have only recently been understood. To date, pertinent studies focused mainly on phenomena related to chromatin structure and DNA methylation, RNAi and other post-transcriptional gene silencing processes, and prions. However, virtually any physiological process can adopt a bistable (or multistable) behavior, as defined by the ability to adopt two (or more) stable states rather than a range of intermediate states, provided that a positive feedback loop exists (or its related counterpart, the mutually inhibitory double negative feedback loop) within the system [1,3,4]. The bistability may sometimes be associated with hysteresis, i.e., the ability of the system to maintain, in a sustained manner, a particular state despite the fact that the stimulus initiating this state is no longer present or is below the level that initially activated the system [5,6]. In some cases, the hysteresis is sufficiently dominant to permit faithful transmission of the different states during mitosis and even meiosis, thus appearing as an epigenetic phenomenon [4,7].

In the light of these concepts, alternative inheritable regulatory states were designated (reviewed in [8]) based on previous descriptions of regulatory or metabolic networks, permitting the definition of new types of inheritance units, the 'toggle switch' and the 'positive feedback switch'. More recently, mathematical models defined the conditions in which a system endowed with positive autoregulation can present bistability [9,10]. However, most studies on bistable regulatory states are presently performed with a few well-known systems [7,8], including the lactose operon, the lambda lysogenic/lytic decision switch, and the *Xenopus *mos MAP kinase cascade. Other relevant models are necessary for the comprehension of these phenomena.

Fungi are an excellent source of well-defined bistable and multistable processes associated with hysteresis (reviewed in [4,11]; see also [12]). In yeasts, such phenomena are quite common and are called phenotypic switches [11,13] or prions [14]. In filamentous fungi, bistable phenomena seem to be particularly prevalent, as one-third of the species show at least one example of these [4]. In most cases investigated in filamentous fungi, bistability results from the appearance and subsequent spread of cytoplasmic and infectious factors. Apart from the HET-s and the Crippled Growth determinants of *Podospora anserina*, their molecular nature and their roles remain unknown. HET-s is a prion [15] and the Crippled Growth determinant seems to result from a positive autoregulation of a signal transduction cascade [16]. The widespread recognition of these infectious factors in fungi is probably due to the ease with which they are detected. The syncytial structure of the mycelium facilitates the propagation of factors from cell to cell. Usually, the presence of the infectious element results in a modification of thallic characteristics, such as fertility or morphology, and is thus easily scored visually as sectors in which the mycelium displays different properties.

**Figure 1:**
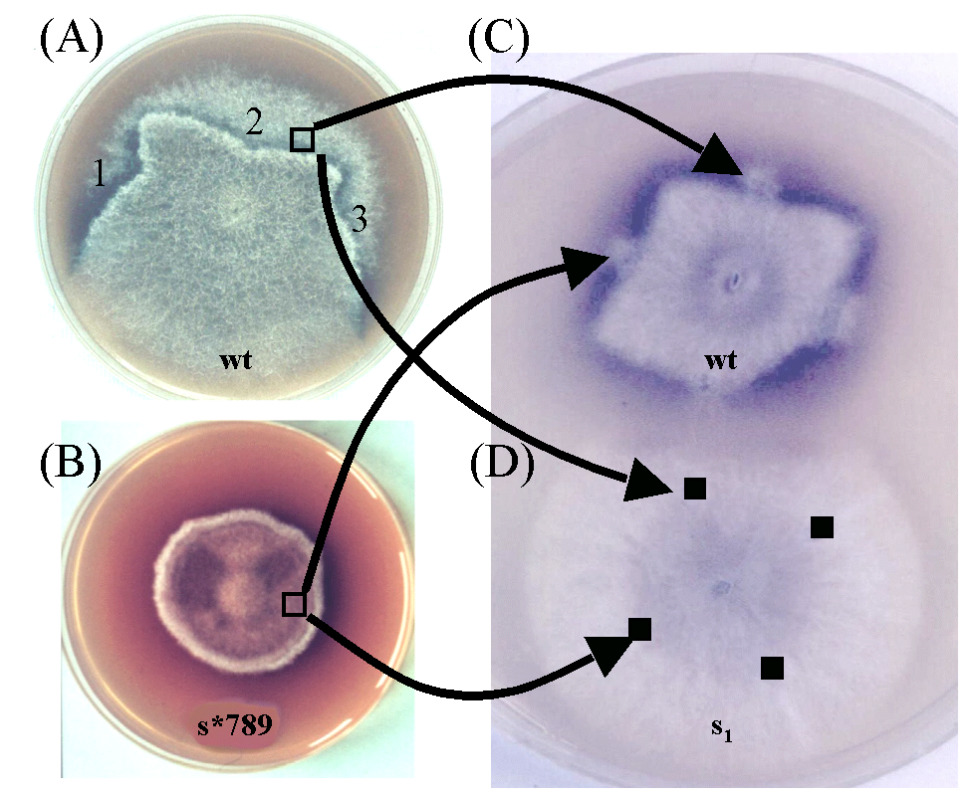
**The Secteur and its transmission through contamination tests in *Nectria haematococca. *(A) **Wild-type strain exhibiting three spontaneous Secteurs (1, 2, 3). **(B) **s*_789 _mutant. **(C) **Contamination tests, using plugs from a Secteur or from s*_789_, on wild type (wt), result in one Secteur at each infection point. **(D)**Contamination tests on *s*_1 _mutant did not result in the induction of any Secteur at the infection points.

In the filamentous ascomycete *Nectria haematoccoca*, two bistable phenomena have been described [17]. *N. haematoccoca *is the teleomorph of *Fusarium solani*, a fungus encountered throughout the world as both saprophyte and pathogen. *N. haematoccoca *produces naphthoquinones exhibiting biological activities. Production of naphthoquinones is regulated by genetic and environmental but also epigenetic factors. The thallus of this species, observed just after ascospore germination, is a dense, grayish aerial mycelium, called Normal. Two kinds of morphological modification (the Anneau and the Secteur) frequently and randomly appear in the growth margin of the thallus as small brown areas in which hyphal elongation is reduced and secretion of pigments is drastically increased (see Figure 1A for the development of the Secteur). Once initiated, the modifications spread and progressively invade the margin of the culture, with a speed that dictates their form (20 times the hyphal elongation speed for the Anneau and twice for the Secteur). The new growth forms can be perpetuated with large pieces of mycelium excised from the growing margin of affected mycelia. The resulting modified subcultures, called ZiS (from a Secteur) and ZiA (from an Anneau), grow slowly and are pigmented in brown. However, fragmentation of a modified culture permits the restoration of the Normal morphology in a variable proportion of subcultures [18], showing that ZiS and ZiA cultures are probably mosaics of infected and uninfected hyphae. Each modification can be specifically transmitted to Normal cultures by contamination experiments, as shown in Figure 1C for Secteurs. The tangential propagation of the new growth forms is thus due to the transmission through hyphal fusion (anastomoses) of specific infectious cytoplasmic determinants, called σ for the Secteur and α for the Anneau.

Mutagenesis experiments of wild-type conidiospores demonstrated that both the Secteur and the Anneau are under the control of nuclear genes [19]. In these early mutageneses, several kinds of mutation were detected. Five mutations prevent the expression of both the Secteur and Anneau, either spontaneously or after inoculation. They differed from wild type in producing more aerial mycelium, lacking pigmentation, and suppressing the sexual stage through self-fertilization. They mapped to at least four loci. Since this type of mutation prevents the generation of both infectious factors, they are now designated *nas *mutants (for 'no Anneau or Secteur').

Several mutations that specifically prevent the formation of Anneaux were also selected [19]. The two different types of mutations recovered, *a *and a*, suggested that the Anneau is under the control of a unique but complex locus. All *a *and a* mutations map to the same locus, *Ans *(for 'Anneau-specific'). Mutants carrying the *a *mutations are unable to generate the α factor, since they never present Anneau morphology and cannot be infected by that factor. Mutants carrying the a* mutations never show Anneaux, spontaneously or after contamination, but display a red color. Surprisingly, when used as donor in contamination with the wild type as recipient, these mutants are able to trigger Anneaux in 100% of attempts, showing that these a* mutants carry the α factor constitutively. It is noteworthy that the phenotype of a* mutants suggests that the mere presence of this factor is sufficient to promote a red color but is insufficient to alter growth.

To date, only one mutation that specifically prevents Secteur formation has been described [18,19]. This s*_789 _(formerly 789) mutant acts similarly to a* mutation in that it entails a red pigmentation (Figure 1B) and specifically prevents the expression of the Secteur, although it possesses the σ determinant throughout the thallus. This property is shown in Figure 1C, by the ability of small inocula harvested in the red s* thallus to induce Secteurs on growing recipient mycelia lacking the σ determinant.

Because of the similar properties of Anneaux and Secteurs, we decided to screen for more mutations that specifically prevent Secteur formation, to determine if *s *mutations (i.e., a mutation that specifically prevents the generation of σ) could be obtained. To this end, we developed novel mutagenesis procedures permitting the recovery of mutations that specifically prevent Secteur development. Most were *nas *mutations. However, a few s* alleles and two *s *alleles (*s*_1 _and *s*_2_) were obtained. Genetic analysis suggested that the σ factor, like the α factor, is under the specific control of a single complex genetic locus. Molecular analysis of the *s*_2 _allele, obtained by insertional mutagenesis, permitted the cloning and characterization of the *Ses *locus. As expected from the genetic data, the locus is complex and encompasses two linked genes, one of which has similarity with putative esterase/lipase/thioesterase enzymes. These data provide evidence that two linked genes could generate a bistable differentiation.

## Results

### Selection and genetic characterization of mutants affected in the expression of the Secteur phenomenon

**Figure 2:**
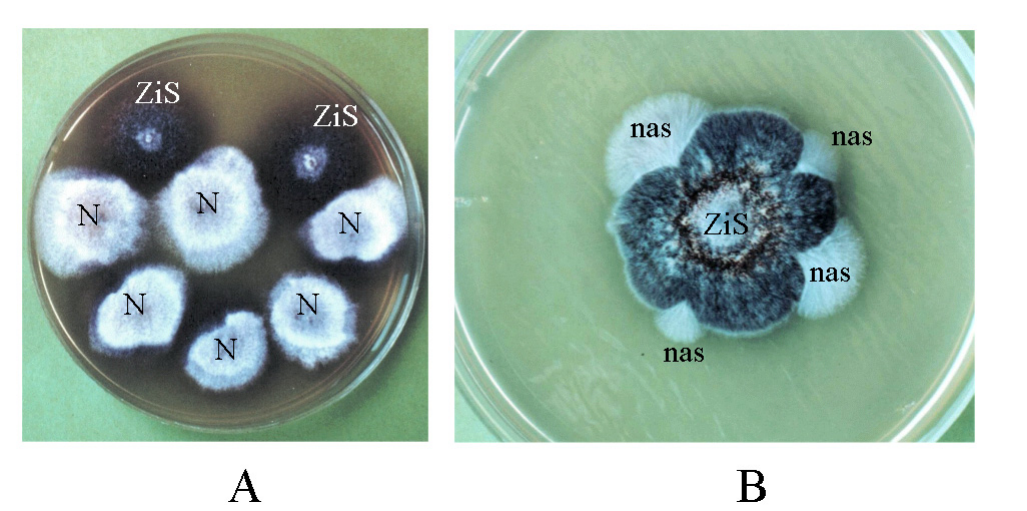
**Selection of *Nectria haematococca *mutants affected in the expression of the Secteur. (A) **Compares the morphology of a Normal growing culture (N) and of a ZiS culture (modified culture obtained by excision from a Secteur). **(B) **A 4-day ZiS culture submitted to UV mutagenesis. Note the emergence of four fast-growing sectors, which correspond to *nas *mutants.

To increase the number of mutants affected in Secteur expression, we decided to use a strategy based on the recovery of fast-growing sectors after mutagenesis of the slow-growing ZiS cultures (see Methods; Figure 2). We recovered 354 independent mutants (Table 1), with a large majority of *nas *mutants (97%). Eleven mutants were specifically affected in Secteur expression. Ten exhibited an s* phenotype identical to that of the s*_789 _strain. One mutant, *s*_1_, had a wild-type phenotype, except that it was unable to express the Secteur, spontaneously or after contamination tests (Figure 1D).

### Genetic analysis of the mutants defines a complex locus involved in Secteur expression

The *s*_1 _mutant and five s* mutants were initially crossed with the *I*_4_*mod *reference strain. All behaved as single mutants, with about 50% wild type and 50% mutant type in the progeny (data not shown). In some progeny, the s* and *s*_1 _mutations were recombined with the *I*_4_*mod *markers. These strains were kept for further crosses. Crossing *s*_1 _with s*_789 _or crossing the new s* mutants with *s*_1 _and with s*_789 _yielded progeny that were mostly segregants, with the parental phenotypes in a ratio 1:1 and few segregants with the wild-type phenotype. Their frequency ranged from 0.1 to 0.6% of the progeny (Table 2), indicating that mutations affecting the Secteur were closely linked. In order to determine dominance/recessivity relationships of the mutated and wild-type alleles, forced heterokaryons were produced by pairing some mutants and wild-type strains marked with auxotrophic mutations (see Methods; Table 3). The *s*_1_/+, s*_789_/+, and s*_84_/+ prototrophic mycelia were grayish and able to differentiate Secteurs, as did the control heterokaryon, which carried only the auxotrophic markers. Thus, the wild-type allele was dominant over its mutated counterparts. Combinations of s* mutants with *s*_1 _gave rise to heterokaryotic wild-type mycelia, indicating that complementation occurred. In contrast, no complementation was observed in pairings involving two different s* alleles.

The simplest interpretation of these results is that *s*_1 _and s* mutations could affect two different linked genes or two separate domains of a multifunctional protein encoded by a large gene. However, due to the low representation of heterokaryotic cells (about 10%) determined in previous studies [20], one must carefully interpret these data, deduced from the characteristics of the prototrophic heterokaryotic mycelium.

### Insertional mutagenesis identified the *Ses *locus

**Figure 3:**
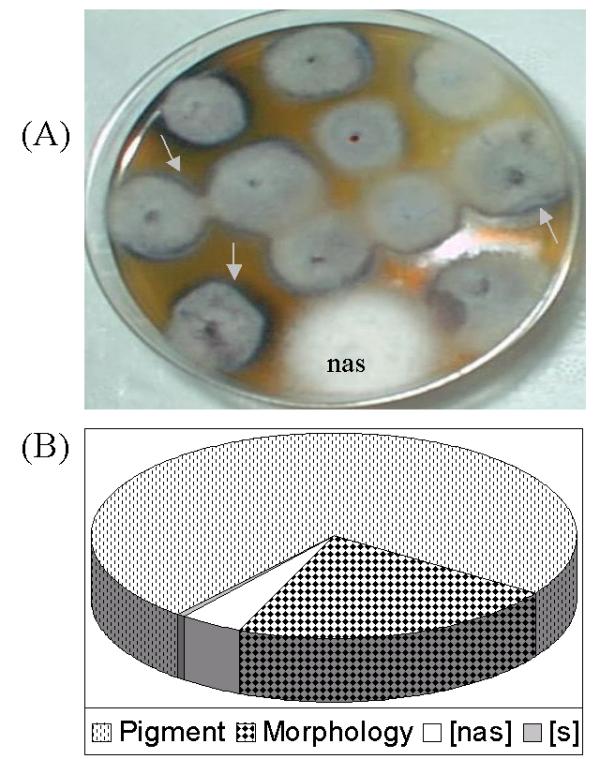
**Phenotypic characterization of transformants. (A) **Detection of a *nas *mutant phenotype among 12 transformants. Arrows indicate some Secteurs. **(B) **Summary of the different mutants recovered among the 5000 transformants: 152 were altered in pigmentation (99 reddish, 16 white/beige/yellow, 37 greenish), 45 were altered in colony morphology (12 irregular, 12 compact, 19 colonial, 2 fluffy), and 10 were affected in the expression of the Secteur (9 *nas *and 1 *s*).

Since the *s*_1 _mutant allele appears recessive to the wild type in balanced heterokaryons, we tried to clone the *Ses *locus by complementation using the sib selection strategy [21]. Although two different cosmid banks, XSG and XN (see Methods), were used, none of the tested transformants exhibited Secteurs. As an alternative strategy, we therefore used insertional mutagenesis, which can rapidly lead to the cloning of the relevant gene. Transformation with a plasmid that carries a hygromycin B resistance marker was performed to identify new mutants affected in *Ses*. However, because the contamination tests necessary for this screen were labor-intensive and time-consuming, we preferred the *61a*_1 _strain as a recipient for transformation, since it expressed Secteurs very early, and at high frequency, and never expressed Anneaux [22]. As illustrated in Figure 3A, the majority of transformants grown for 15 days at 18°C differentiated Secteurs, so that few transformants had to be tested by contamination experiments for their Secteur expression. During the course of these experiments, we also screened transformants harboring a variation in colony morphology or pigmentation (Figure 3B). These phenotypes could be maintained from conidia isolation, showing that the transformation procedure was an effective approach in the creation of mutants. Ten transformants among the 5000 recovered failed to express the Secteur. Nine exhibited a *nas *phenotype. One was probably affected in the *Ses *locus, as it was unable to differentiate Secteurs, either spontaneously or after contamination tests. This transformant, called *s*_2_, had a wild-type morphology and behaved as did the *s*_1 _mutant previously isolated.

### The *s2 *transformant carries a DNA fragment integrated in the *Ses *locus

In order to prove that *s*_2 _carries integration in the *Ses *locus, we crossed it with the wild-type (wt) and *s*_1 _strains. When crossed with wt, the 100 tested ascospores segregated 1:1 for ability:inability to differentiate Secteurs. As expected, hygromycin B resistance always cosegregated with the inability to differentiate Secteurs. When *s*_2 _was crossed with the *s*_1 _strain, only seven ascospores among 1657 were unable to express the Secteur (Table 2), indicating that *s*_2 _also mapped at the *Ses *locus. Overall, the data strongly suggested that *s*_2 _resulted from a single insertion at the *Ses *locus.

**Figure 4:**
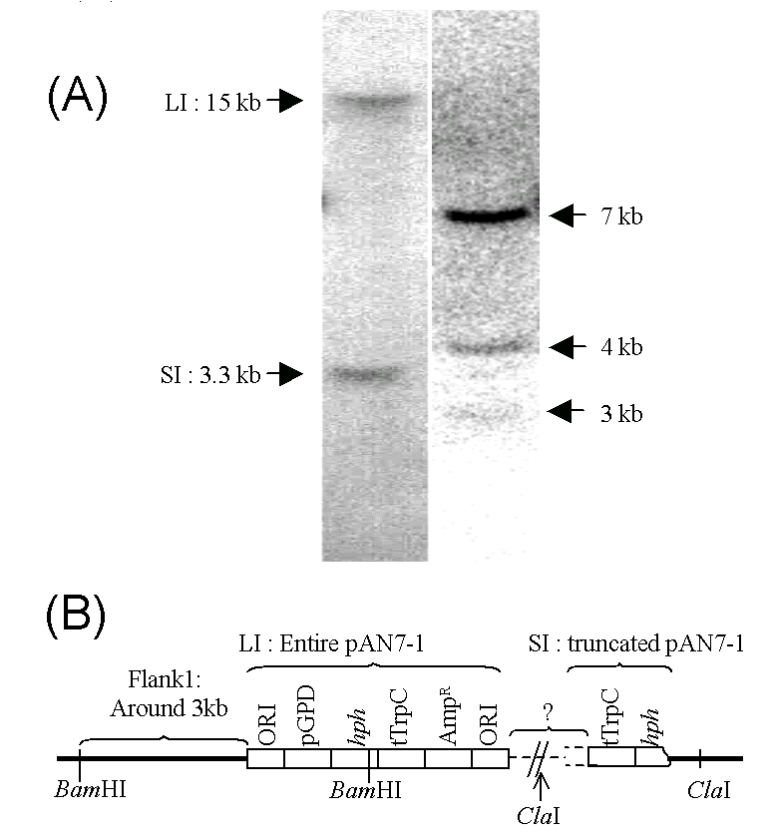
**Analysis of pAN7-1 integration pattern in *s*_2_.(A) **Southern blot analysis of total genomic DNA of *s*_2 _digested with *Cla*I, which does not cut the plasmid (lane 1), and *Bam*HI, which cuts once in pAN7-1 (lane 2), and probed with the pAN7-1 vector. Sizes are indicated in kilobases (kb). **(B) **Restriction map of the pAN7-1 integration point in *s*_2_. Wild-type genomic DNA is shown as a thick line, pAN7-1 sequences are represented by the open bar, and the dotted line stands for unknown sequences. The fragment LI is inserted at the bacterial replication origin (ORI). SI is truncated and carries a part of *hph *and of *tTrpC*.

To establish the integration pattern in *s*_2_, Southern blotting analysis was performed after digestion by *Cla*I, which did not cut the plasmid, and by *Bam*HI, which cut once in the vector. The probe was the pAN7-1 vector used for insertional mutagenesis. As shown in Figure 4A, the number and intensity of hybridizing bands in *Cla*I digest reflected a complex integration event, with at least two integrated copies. Southern blot analysis of 10 *s *progeny from an *s*_2 _× wild-type cross did not show segregation of the two integration sites (data not shown), suggesting that the two integrated copies were linked. The size of the larger *Cla*I-fragment, LI, was compatible with an entire copy of the vector, whereas the smaller, SI, could be interpreted as a truncated copy. Further analyses with other restriction enzymes and probes (data not shown) established the restriction map of this region (Figure 4B). The sequence of a region flanking SI revealed that both LI and SI occurred at the same genomic site and were separated by a DNA fragment of unknown origin (Figure 4B).

### Molecular cloning of the *Ses *locus

**Figure 5:**
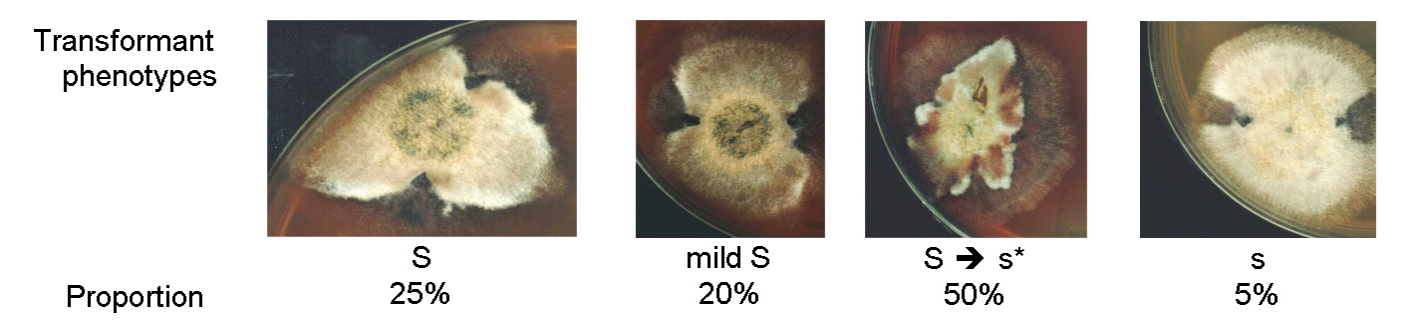
Types of transformants recovered in transformation experiments using s* mutants with XN31E6 and psecX5. As the respective proportion of each type of transformant was similar with both vectors, the proportion observed is the combined percentage computed with 60 tested transformants. S, transformants differentiating Secteurs as wild type; mild S, transformants differentiating a Secteur that propagates more slowly than wild type and that frequently reverts to a normal morphology. S → s*, transformants differentiating the Secteurs but showing an s* phenotype beyond the modified area; s, transformants unable to differentiate Secteurs.

**Figure 6:**
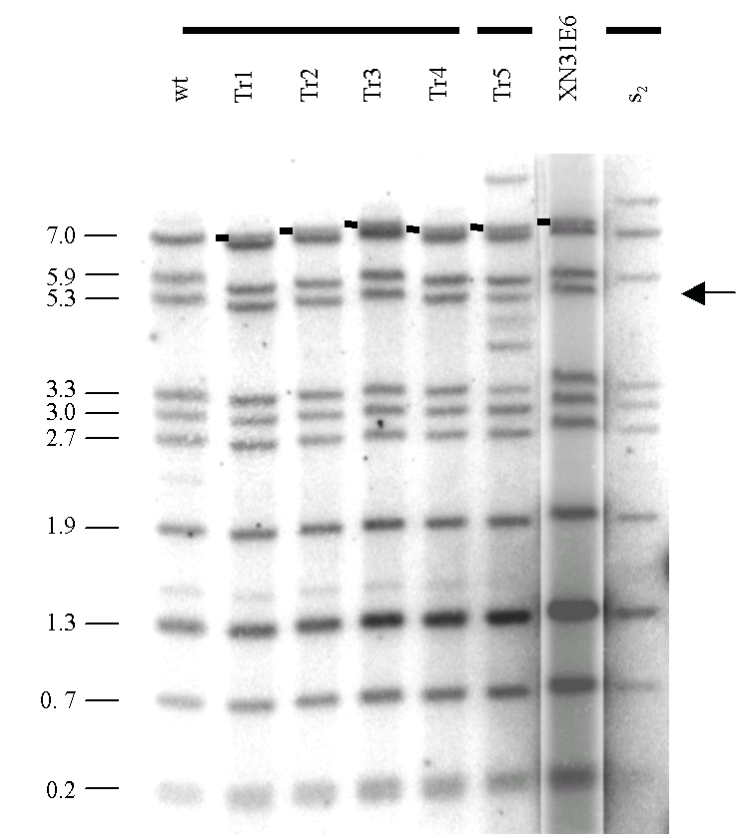
**Southern blot analysis of XN31E6 transformants. **XN31E6 cosmid DNA along with total genomic DNAs of wild type (wt) *s*_2_, and five transformants obtained by transforming s*_789 _with XN31E6 were digested by *Xho*I and hybridized with an XN31E6 probe. Wild type, Tr1, Tr2, Tr3, and Tr4 differentiate wild-type Secteurs (S), Tr5 has a mild S phenotype, and *s*_2 _is not able to differentiate Secteur(s). The 7-kb band corresponding to pMoCosX is marked by dark connecting lines. The arrow points to the 5.3-kb fragment, which shifts in *s*_2_.

The restriction enzyme *Bam*HI was used to recover the genomic sequence flanking LI (Flank1 in Figure 4B) along with part of the integrated pAN7-1 vector. A part of Flank1 was then used as a probe to screen the XN cosmid library. One hybridizing cosmid, XN31E6, was identified and used to transform the s*_789_, s*_18_, s*_27_, and s*_4 _mutants. Most of the transformants recovered the ability to express the Secteur, showing that the s* mutations are probably recessive. However, different phenotypes were observed (Figure 5). The 'mild S' exhibited the Secteur altered in its propagation and maintenance, and the 'S → s*' showed an s* phenotype beyond the Secteur. The mild S was interpreted as having a reduced amount of σ in the modified areas because of insufficient expression of the transgene, and the S → s* type was probably due to transgene sorting. Indeed, wild-type and s* nuclei could be recovered through the isolation of microconidia in such transformants, before expression of a Secteur, indicating that the S → s* thalli had a heterokaryotic structure. After the development of a Secteur, only the s* cells that were not affected in their growth in the presence of σ could grow, as previously observed for balanced heterokaryons [20]. Interestingly, we also observed several transformants displaying an s phenotype (Figure 5). These might have resulted from an abnormal integration inactivating the *Ses *locus. Molecular analysis of five hygromycin-resistant (Hyg^R^) transformants (four with a wild-type phenotype, and one with a mild-S phenotype) revealed two patterns of integration (Figure 6). The pattern exhibited by the four wild-type transformants was wild type, except for an additional band corresponding to the pMoCosX vector. These transformants were thus interpreted as having integrated one copy of XN31E6 at the resident *Ses *locus. The mild-S transformant pattern was more complex, showing additional bands and a difference in fragment stoichiometry, suggesting a complex integration. This is consistent with a reduced amount of σ hypothesized for mild-S transformants.

It is noteworthy that despite several attempts, transformation experiments done with the *s*_1 _mutant as a recipient did not yield any transformants able to express the Secteur. This suggests that, contrary to the observations in balanced heterokaryons, *s*_1 _is dominant in partial diploids. Dominance of *s*_1 _was indeed established through transformation (see below). This dominance in diploids explains the failure to clone *Ses *by complementation.

**Figure 7:**
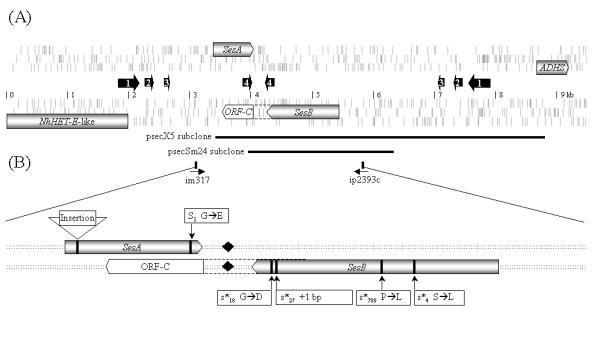
**Schematic representation of the *Ses *locus of *Nectria haematococca. *(A) **Open reading frames larger than 200 aa or showing sequence similarity to other genes are represented by gray arrows. Black arrows represent sequences in inverted repeated orientation: repeat 1 is 290 bp long; repeat 2 is 79 bp long; repeat 3 is 32 bp long; and repeat 4 is 93 bp long. Thick lines: subclones able to restore the Secteur modification in s* mutants. **(B) **Detailed map of the region between oligonucleotides im317 and ip2393c. Vertical bold lines: position of the indicated mutations. Insertion: integration point of pAN7-1 in *s*_2_. Black lozenge: polyadenylation site.

In order to further define the DNA fragment complementing the recessive s* mutation, cosmid XN31E6 was subcloned into pBluescript and the plasmids were assayed for complementation. This yielded psecX5, a plasmid carrying a 5.5-kb *Xho*I fragment sufficient to confer Secteur expression (Figure 7) with the same efficiency as did XN31E6.

### Gene organization of the *Ses *locus

A 9-kb-long region surrounding the integration site in the *s*_2 _strain was sequenced (GenBank Accession no. AY572411). One interesting feature of the sequenced region concerned the presence of several inverted repeats ranging from 32 to 290 bp and organized in a symmetrical fashion (Figure 7). Inspection of the sequences revealed four open reading frames (ORFs) that were either larger than 200 amino acids or showed significant similarity with proteins in the databases (Figure 7). To begin with, we found an ORF encoding a putative large protein of unknown function that is also detected in the complete genome sequences available for the other filamentous ascomycetes (those of *Neurospora crassa*, *Magnaporthe grisea*, *F. graminearum*, *Aspergillus nidulans*, *Aspergillus fumigatus*, and *P. anserina*). This protein, NhHET-E-like, has a weak similarity (23% identity and 39% similarity over 455 amino acids) to the HET-E protein of *P. anserina, *which has been shown to be involved in heterokaryon incompatibility [23]. On the other side of the insertion site, a partial ORF of 165 amino acids carrying a PROSITE (PS00061), short-chain alcohol dehydrogenases/reductases family signature, was detected and designated ADHS. Two additional ORFs, *SesA *and *SesB*, were also identified. Functional analysis revealed that only these two ORFs were part of *Ses, *each having a different role.

**Figure 8:**
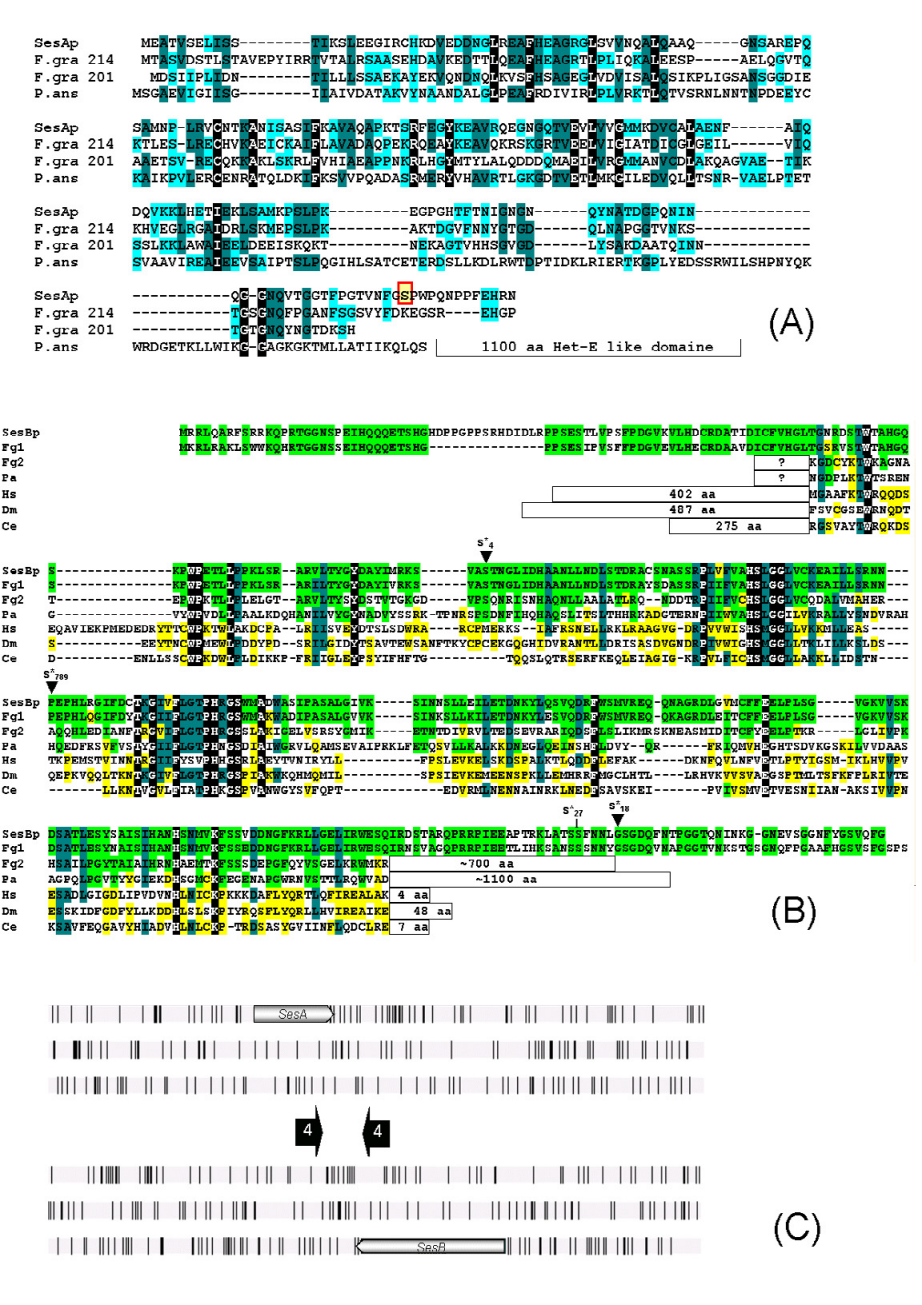
***SesA *and *SesB *conservation during evolution. (A) **Alignment of SesAp with its homologues in *Fusarium graminearum *(F. gra 214 and F. gra 201) and *Podospora anserina *(P. ans). The amino acid change in *s*_1 _is boxed. **(B) **Alignment of SesBp with representative homologues from *F. graminearum *(Fg1 and Fg2; the sequence was obtained from the Fungal Genome Initiative, ), *P. anserina *(Pa; ), *Homo sapiens *(Hs; GENBANK: BC001705), *Drosophila melanogaster *(Dm; GENBANK: AAF5369), and *Caenorhabditis elegans *(Ce; PIR: T21079). Arrowheads indicate the amino acid changed in s*_4_, s*_789_, and s*_18_; arrow indicates the position of the frameshift in s*_27_. **(C) **Conservation of Ses organization in *F. graminearum*. *SesA *corresponds to F. gra 214 of **(A) **and *SesB *corresponds to Fg1 of **(B)**. Percentages of identity with *Nectriahaematococca *SesAp and SesBp are 41% and 79%, respectively. In **(A) **and **(B)**, conserved amino acids are shaded. Color reflects the degree of conservation.

*SesA *is a 210-codon ORF (Figure 7) with no homologue in the genomes of *N. crassa*, *M. grisea*, *A. nidulans*, or *A. fumigatus*. However, it is significantly similar (30% identity and 42% similarity) to the N terminus of a large putative protein of *P. anserina *of unknown function but with 46% identity and 60% similarity with the Het-E protein in its C terminus (Figure 8A). It is also similar to two ORFs, of 214 and 201 codons, present in the genome of *F. graminearum*, with 41% identity and 54% similarity over 197 amino acids and 32% identity and 49% similarity over 199 amino acids, respectively (Figure 8A). *SesA *is interrupted by pAN7-1 in the *s*_2 _transformant, and sequence analysis of *SesA *in the *s*_1 _mutant showed a mutation at the end of the coding sequence, which changes a glycine into a glutamic acid (Figures 6, 7, 8A). *SesA *was expressed because a 480-bp-long RT-PCR product was obtained by using the 3' RACE (rapid amplification of cDNA ends) procedure. The sequence of this product revealed that a polyadenylation signal existed 141 bp downstream from the *SesA *stop codon.

*SesB*, a 386-codon ORF (Figure 7), is located on the opposite strand from *SesA*. *SesB *shows significant similarity with several putative proteins from filamentous ascomycetes (at least three in *A. fumigatus *and *A. nidulans*, six in *F. graminearum, *two in *N. crassa*, and four in *M. grisea *and *P. anserina*) and animals (*Caenorhabditis elegans*, *Drosophila melanogaster*, or mammals). It does not show similarity with hemiascomycetous yeast ORFs (*Saccharomyces cerevisiae *and 12 other partially sequenced yeasts), basidiomycete ORFs (*Coprinus cinereus*, *Ustilago maydis*, and *Phanerochaete chrysosporium*), or *Arabidopsis thaliana *ORFs. All the proteins similar to *SesB *show a significant PROSITE (PS50187) esterase/lipase/thioesterase active site serine domain. The sequence of *SesB *in *s*_1_,s*_789_,s*_4_,s*_27_, and s*_18 _revealed that in the four s* mutant alleles, *SesB *contains a single point mutation (Figures 7 and 8B), but no mutation in the *s*_1 _mutant. In s*_789_, s*_18_, and s*_4_, the mutations are missense mutations, and in s*_27_, the mutation corresponds to a one-base addition resulting in a frameshift 120 bp upstream of the stop codon. None affected highly conserved residues of the esterase/lipase/thioesterase domain, suggesting that a functional protein was expressed in the mutants. The expression of *SesB *was also investigated with the 3'-RACE procedure. A 370-bp-long RT-PCR product was obtained, confirming that *SesB *is expressed. Sequencing showed that a polyadenylation signal is used 81 bp downstream from the *SesB *stop codon. Downstream of *SesB *is another potential short ORF (ORF-C), encoding a putative peptide of 157 amino acids with no homologue in the databanks. To show that ORF-C is not an exon of *SesB*, we cloned into pBluescript the 2.4-kb-long *Sma*I fragment carrying only *SesB*, i.e., without ORF-C or *SesA*. This yielded plasmid psecSm24 (Figure 7). Its transformation in four different s* mutants showed that the insert restored the expression of the Secteur. The molecular analysis of transformants showed that the integration was ectopic (data not shown), supporting the hypothesis that *SesB *alone is per se a functional gene involved in Secteur development.

Since homologues of both *SesA *and *SesB *are present in the genomes of *F. graminearum *and *P. anserina*, we investigated whether the gene arrangement was conserved in these two species. In *P. anserina, *for which a single *SesA *homologue is found, the gene arrangement is not conserved, since *SesA *and *SesB *homologues are on two different contigs. In *F. graminearum*, the putative 201-amino-acid homologue of *SesA *that displays the lowest percentage of identity is not associated with any of the *SesB *homologues. On the contrary, the 214-amino-acid homologue of *SesA *that displays the highest identity is associated with a *SesB *homologue in the same order (Figure 8C). This homologue is highly conserved between *N. haematococca *and *F. graminearum *(79% identity and 85% similarity over 387 amino acids) whereas the other *F. graminearum *homologues are less conserved (40% identity and 63% similarity over 273 amino acids for the next-best homologue). In *F. graminearum*, the inverted repeats 1, 2, and 3 are absent but the inverted repeat 4 is present. Overall, these data suggest an evolutionary conservation of *Ses *between *N. haematococca *and *F. graminearum*, both of which are in the genus *Fusarium*, but with a lack of conservation of the gene organization in more distant species, including the loss of *SesA*. There are no data concerning the potential development of Secteur in the *F. graminearum *strain used by the Fungal Genome Initiative for sequencing.

### *s1 *is dominant in diploids

**Figure 9:**
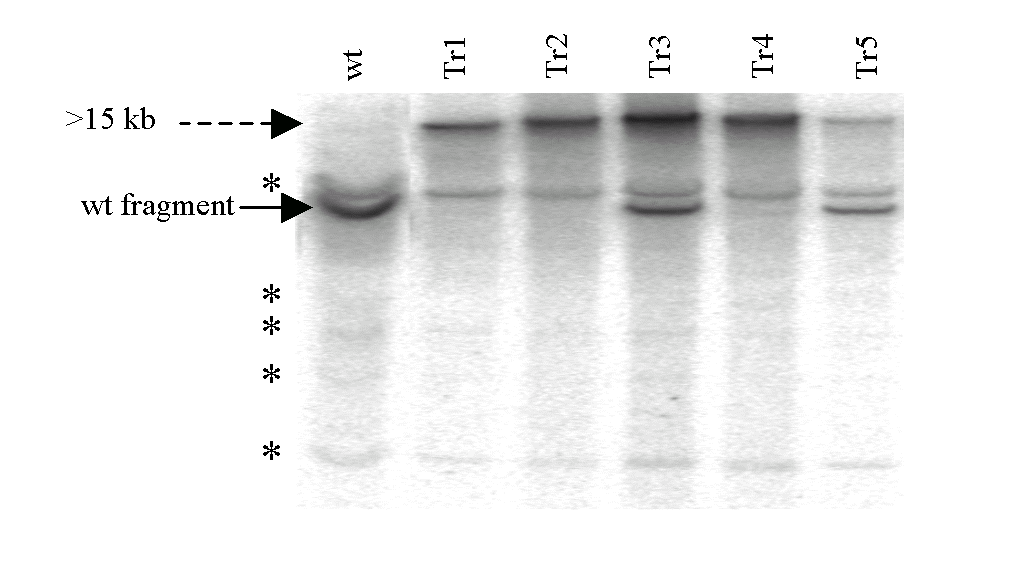
**Southern blot analysis of *Stu*I-digested DNAs of wild-type *Nectria haematococca *and five transformants. **Total genomic DNAs of wild-type (wt) *Nectria haematococca *and five *s *transformants (Tr1-Tr5) that were obtained after co-transformation of wt with psec3-s_1 _plasmid and pBC-Hygro vector, and probed with psec3-s_1_. All the transformants share a large band (>15 kb), absent from wild type. Tr3 and Tr5 contain both the wild type and the *s*_1 _allele. The faint bands noted * correspond to repeated sequences homologous to those present at the *Ses *locus.

To investigate the dominance of *s*_1_, we co-transformed the wild-type strain with pSec3-s_1 _and pBC-Hygro. pSec3-s_1 _was obtained by cloning into 'pGEM-T easy' (Promega, Charbonnières, France), a PCR amplification product constructed with oligonucleotides im317 and ip2393c (see Figure 7) using the s_1 _mutant genomic DNA as template. Thus, pSec3-*s*_1 _contained the entire *Ses *locus present in the s_1 _mutant strain. Among 17 tested Hyg^R ^transformants, seven could not differentiate Secteurs. *Stu*I-restricted DNA from five of these transformants was analyzed by Southern blotting. Two integration patterns were observed (Figure 9). For three transformants, the expected wild-type *Stu*I fragment was absent, suggesting that integration altered the *Ses *locus, thus accounting for the lack of Secteur formation. For the two other transformants, the wild-type *Stu*I fragment was present, as was an additional band larger than 15 kb. As this band hybridized with psec3-s_1 _and the *hph *hygromycin resistance marker (data not shown), these transformants could be interpreted as bearing a co-integration of pBC-Hygro and pSecs_1 _at an ectopic position. Sequencing of the *Ses *locus in these two transformants that do not display Secteurs confirmed the presence of both alleles, i.e., the wild-type allele at the resident locus and the mutated allele *s*_1 _at an ectopic position. This clearly demonstrated the dominance of the *s*_1 _mutation, in contrast to the s* mutations, which are recessive.

## Discussion

Although the Secteur and Anneau phenomena were described about 30 years ago [17], the molecular mechanisms able to generate these fascinating bistable differentiations are still mysterious. Early studies suggested peculiar epigenetic mechanisms [24]. In this study, we have initiated the molecular characterization of *Ses*, the primary locus controlling the development of Secteurs.

### The impact of mutagenesis procedures

The experimental design, based on the differences in the growth rate between modified and normal cells, was very efficient in finding mutations that block the propagation of Secteurs after the action of both UV and NG (*N*-methyl-*N*'-nitro-*N*-nitrosoguanidine). A vast collection of mutants affected in the Secteur expression was easily obtained. One mutant with an *s *phenotype was isolated, along with 11 s* mutants. Apparently, NG (*N*-methyl-*N*'-nitro-*N*-nitrosoguanidine) mutagenesis was more efficient than UV mutagenesis in recovering these specific mutants (9/105 and 3/250, respectively). Genetic analysis showed that the structure of the *Ses *locus mirrors the structure of the *Ans *locus, indicating that α and σ could be generated through similar mechanisms, in agreement with the fact that both factors require the same set of *nas *genes for propagation.

### Relationships between *s1 *and wt alleles: recessivity in heterokaryons versus dominance in partial diploids

Dominance/recessivity tests using balanced heterokaryons showed that *s*_1_/wt heterokaryons were able to differentiate Secteurs as wild type. The recessivity of *s*_1_, deduced from the heterokaryon tests was the initial point of our cloning strategy, i.e., the use of cosmid libraries from wild type to restore the ability to express the Secteur in an *s*_1 _recipient strain. The screening of two representative cosmid libraries failed, and we examined various hypotheses to explain this failure, especially the possibility of the dominance of *s*_1_. In order to easily clone the *Ses *locus, we used an insertional mutagenesis strategy, a powerful method for gene isolation in filamentous fungi [25]. With the pAN7-1 vector, 5000 Hyg^R ^transformants were screened for defects in mycelium pigmentation, colony morphology, and the expression of the Secteur and around 4% of mutant phenotypes were detected. This frequency is similar to the 0.4 to 1.4% reported for loss of pathogenicity in different fungal species [25]. Among them, 9 *nas *mutants and 1 *s *mutant were obtained. Given the proportion observed for classical mutagenesis (12 s* or *s *mutants among 355 *nas*), the recovery of the *s*_2 _mutant was an unexpected outcome. Although we may have been lucky, the recovery of this transformant may reflect a preference for plasmid integration in the region of the *Ses *locus, which is compatible with the high recombination frequency observed at *Ses*. Cloning of *Ses *permitted the construction by transformation of a partial diploid containing both the wild-type and *s*_1 _mutant alleles. The fact that this partial diploid has a *s *phenotype indicates that *s*_1 _allele is indeed dominant over the wild-type allele in partial diploids. Similar contradictions have been observed in *A. nidulans *by comparing diploids and heterokaryons for complementation between mutations in the regulatory and the cognate structural genes. These data were interpreted as being due to a limitation of regulatory gene products to the nucleus or by a stringent dose effect when combined with a nonrandom distribution of nuclei in heterokaryons [26,27]. In our study, a dose effect is also probable. In *N. haematococca*, as reported earlier [20], only 1–10% of hyphal fragments containing 3–4 cells are heterokaryotic, the others corresponding to both homokaryons in similar proportions. In addition, phase-contrast microscopic observation of the prototrophic mycelia revealed the presence of rows of uninucleate cells disrupting the heterokaryotic association. This structure does not prevent the stability of balanced heterokaryons with regard to metabolic requirements (hence the complementation of the auxotrophic mutations), but might severely affect the dosage of the *SesA *products expressed from the wild-type and *s*_1 _nuclei in different portions of the thallus. Because *s*_1 _is dominant and corresponds to a missense mutation, it is likely that the product expressed from *SesA *in *s*_1 _is a dominant negative. If its concentration is insufficient, especially in hyphae homokaryotic for wild-type *SesA *nuclei, the propagation of σ and Secteur formation is favored. In contrast, in diploids an even ratio of the two products is always achieved, preventing propagation of σ and Secteur formation.

### Integrated model of functional elements at the *Ses *locus

The molecular characterization of *Ses *shows that it is composed of two linked and expressed genes, *SesA *and *SesB*, both of which are necessary for Secteur expression. Each has a different role. The *s*_1 _and *s*_2 _mutants map to *SesA*. Because *s*_2 _is an integration of a large segment of DNA at the beginning of *SesA, *the phenotype of the *s*_2 _mutant probably results from a complete loss of function of *SesA*. The *s*_1 _mutant carries a dominant missense mutation and therefore synthesizes the *SesA *mRNA, and probably a dominant negative protein. The SesAp protein has no evident homologue in the databanks yielding a clue to its function. As *s*_2 _does not produce the σ determinant, cannot be contaminated by mycelia carrying σ, and never differentiates the Secteur morphology, *SesA *is probably necessary for the production and transmission of σ.

All s* mutations map to *SesB *and determine a red-pigmented phenotype associated with the generation of the σ determinant throughout the thallus, but without any growth alteration. It is noteworthy that all the s* mutations are probably not simply preventing the production of a functional mRNA, but are also not preventing the production of an active protein, as they map outside the evolutionarily conserved region of the protein. The function of *SesB *and its homologue remains unknown in fungi; however the inactivation by RNA interference of the most similar gene in *C. elegans *is lethal during embryogenesis, suggesting that the protein produced by *SesB *could be essential (accession NP_510177; [28]). Based on the analysis of the mutant phenotype triggered by s* mutation, the wild-type *SesB *product is involved in the repression of pigmentation and σ generation.

To date, this situation has not been described for any of the previously reported systems responsible for bistable or multistable switches in fungi. Previous systems implicated prions [23,29], chromatin silencing [12], hysteresis in a MAP kinase cascade [16], and possible membrane inheritance [30,31]. At present, it is premature to propose a molecular model to explain the Secteur, because *SesA *and *SesB *display weak similarity only with proteins of unknown function. However, from our data we can infer that *SesA *most likely encodes a regulatory factor for *SesB *and that the regulation probably takes place at the protein level and probably not at the RNA level, since *s*_1 _and all s* mutation are point mutations that are not likely to affect RNA transcription and stability. Two formal models could explain how the regulation might work.

**Figure 10:**
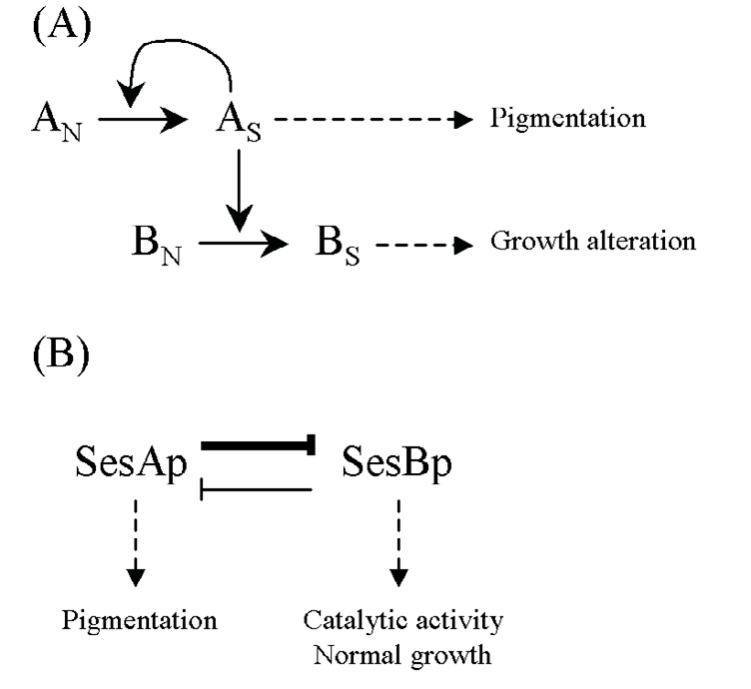
**Models integrating functional elements of Secteur expression in *Nectria haematococca. *(A) **First model: In the wild-type strain, the product of the *SesA *and *SesB *genes can exist in a normal state (A_N _and B_N_) and in a modified state (A_S _and B_S_). A_S _determines overproduction of pigments and B_S _is responsible for growth alteration. **(B) **Second model: The product of *SesA*, SesAp, is responsible for pigmentation. The product of *SesB*, SesBp, has a catalytic activity necessary for the normal growth. These two products negatively regulate each other, and the equilibrium constants favor SesAp. In juvenile thalli, only SesBp is produced, but SesAp can be randomly produced, switching to a new state with a majority of SesAp.

Firstly, the product of *SesA *(SesAp) could exist in two states: one, A_N_, characterizing the Normal morphology and the other, A_S_, determining the Secteur morphology (Figure 10A). The transition A_N _to A_S _may be initiated by a rare event in cells of the growing margin of the thallus. Once formed, the A_S _state becomes predominant by directing other A_N _molecules to adopt the same state. This positive feedback loop allows A_S _propagation from hyphae to hyphae through anastomoses. A_S _alone would induce the hyperproduction of pigments (hence the absence of both σ and pigmentation in the *s *mutants, as they are mutated in *SesA*). In addition, the product of *SesB *(SesBp) can adopt the normal B_N _state, which can be transformed into a B_S _state in the presence of A_S_. B_S _would be responsible for the growth alterations observed in the Secteur and would follow the propagation of A_S _(hence the lack of growth impairment presented in the s* mutants, as they are mutated in *SesB*).

In the second model, SesAp may negatively regulate SesBp but also be negatively regulated by SesBp (Figure 10B). In the normal mycelium, SesBp, which probably has a catalytic activity, would be active in promoting healthy growth. The appearance in some cells from the growing margin of a sufficient amount of SesAp would displace the equilibrium, resulting in the inhibition of SesBp. In order for this to happen, the equilibrium constants must be in favor of SesAp. This would result in growth impairment (because SesBp would not be present) and in a red pigmentation (because SesAp would be present). In the *s *mutants, SesAp would never be present, thus making the presence of SesBp constitutive (hence the absence of Secteurs and the healthy growth). In the s* mutant, the interactions between the two proteins would be abolished, permitting the activity of both SesAp and SesBp (hence the presence of a constitutive σ, the red pigment, and healthy growth). This model could explain the dynamic equilibrium found in the ZiS area, as well as the dose effect for SesAp, explaining the difference of heterokaryons and partial diploids.

## Conclusions

Secteur, one of the two bistable and hysteretic differentiations exhibited by the filamentous fungus *N. haematococca*, is controlled by a complex, two-gene locus. Each gene has a distinct role in defining bistability, and atypical regulatory relations between the two proteins coded by the two genes may account for the hysteresis of Secteur differentiation. The data exemplify the diversity of mechanisms that generate differentiation in fungi, and, more generally, in eukaryotes.

## Methods

### Fungal strains and growth conditions

The homothallic *N. haematococca *strain wt (Centraal Bureau voor Schimmelcultures, Baarn, The Netherlands) was used as the standard wild-type strain in this study. The origin and relevant characteristics of all strains used in this study are listed in Table 4.

The a_1_s_1_I_4_mod and 61a_1 _strains used as recipients in transformation experiments were constructed by crossing the s_1_I_4_mod strain with a_1 _mutant and the 61 mutant with a_1_I_4_mod strain, respectively (unpublished data). The marker segregation was recorded after three weeks. This included (1) mycelium and perithecium pigmentation, (2) fertility, and (3) the ability to express the Secteur and the Anneau as tested by transferring the progeny onto potato dextrose agar (PDA) and by inoculating 3-day cultures with s*_789 _(for Secteurs) and a*_58 _(for Anneaux).

General culture conditions and manipulations were as described by Daboussi-Bareyre [20]. Strains were purified through microconidia and maintained on PDA at 26°C. Long-term stocks were stored as plugs under mineral oil at 12°C. The medium selective for transgenes was PH8, i.e., PDA containing 8 μg/ ml of hygromycin B from Sigma-Aldrich (Saint Quentin Fallavier, France).

### Mating and ascospore recovery

The general methods for crossing the homothallic *N. haematococca *have been described elsewhere [19,32]. Since the strains used in this study were homothallic, detection of hybrid perithecia in crosses were performed using as a partner in crosses the double mutant strain I_4_mod. This strain developed white, self-sterile perithecia whereas wild-type and fertile mutants developed self-fertile, red perithecia [32]. Thus, all the white, fertile perithecia, which appeared on the crossing plates, were hybrid.

The progeny of crosses was analyzed in accordance with the following procedure: two to nine white, fertile perithecia from each cross were freed from mycelium and conidia. Each was opened to spread the ascospores on one Petri dish containing water plus 3% agar. To control viability, 35 ascospores from each perithecium were transferred to PDA and incubated for 4 days at 26°C, while the water-agar stock dishes were kept at 2°C to prevent ascospores from germinating. Then, the water-agar dishes containing many viable ascospores (usually those with more than 70% of germination were selected) were returned to 26°C to allow germination overnight. A sample of about 400 germinating ascospores from each dish was transferred to PDA. The phenotypes were recorded after a week at 26°C in the dark, except for the perithecium color, which was evaluated after an additional period of two weeks.

### Heterokaryon test

Heterokaryons were constructed as described in [20]. Initially, strains s_1_, s*_84_, and s*_18 _were crossed with the two strains carrying either of the markers, arg_154 _and lys_255_, that promote auxotrophy for arginine and lysine, respectively. Auxotrophic marked strains, which were recovered in the progeny, were paired on a cellophane membrane in the combinations appropriate to restore prototrophy. After 48 hours of growth on complete medium, the membrane was transferred to minimal medium. Widely inclusive inocula from prototrophic mycelia observed at the junction of the two partners were transferred to minimal medium and examined for their pigmentation and their ability to express the two modifications, either spontaneously or after inoculation.

### Standard mutagenesis

Plugs taken from the growing margin of a culture modified by the Secteur were inoculated on cellophane discs on PDA and grown for 4 days at 26°C. The modified slow-growing cultures (ZiS) were submitted to mutagenesis by UV or NG. For UV mutagenesis, cultures were exposed at 450 J/m^2^, with a UV light source (254 nm), and then kept in the dark for 72 hours. For NG mutagenesis, 0.1 ml of a 200γ/ml solution of NG was deposited under the cellophane disc. After 1 hour, the treated cultures were exposed to light for NG degradation.

Fast-growing sectors were clearly visible a week after mutagenesis. For the estimation of the number of sectors recovered from each treated thallus, only sectors harboring different phenotypes were considered. A plug of these independent sectors was inoculated on PDA and the resulting cultures were examined for pigmentation and ability to express the Secteur and/or the Anneau at 26°C. The majority of the recovered mutants corresponded to *nas *mutants, defined by (1) the inability to differentiate both Anneaux and Secteurs, (2) an exuberant white mycelium, and (3) the inability to differentiate perithecia. Some of these mutants could express the modifications at other temperatures, as was previously observed for mutants 727 and 100 [33,34]. s* mutants were recovered as red, fast-growing sectors. They displayed the same properties as the s*_789 _mutant described previously [19], i.e., red thallus and inability to express the Secteur spontaneously or after inoculation. A unique *s *mutant was selected as a wild-type-growing sector. This mutant displayed a wild-type phenotype, except for the inability to express the Secteur spontaneously or after inoculation. Both s* and *s *mutants could differentiate Anneaux.

### Insertional mutagenesis

Plasmids pAN7-1 (GenBank accession number Z32698) or pBC-Hygro [35], which had no sequence similarity with the *N. haematococca *genome, were used to transform the double mutant strain *61a*_1_. Both vectors carried a hygromycin resistance gene. Transformation occurred at a frequency of about 10 transformants/μg plasmid. Southern analysis on 10 independent hygromycin-resistant transformants (Hyg^R^) indicated that the transforming DNA inserted generally at one or two genomic sites, in some cases in a tandem fashion (data not shown). This pattern of integration was suitable for recovery of tagged genes.

To screen for mutant phenotypes, 5000 Hyg^R ^independent transformants were transferred to PH8 (12 per Petri dish) and grown for 15 days at 18°C, a temperature that increased the frequency of spontaneous Secteur formation. Thalli with mutant phenotypes were frequently recovered (Figure 3). These most likely resulted from the plasmid integrations, since a sample of regenerating protoplasts treated in the same way but without the transforming DNA did not show any phenotypic variation. As control, a sample of transformants with an altered morphology was purified through single conidial isolation. Data showed that the mutant phenotype was stable. Potential candidates affected in the expression of the Secteur were recovered as thalli that did not form spontaneous Secteurs. Inoculation experiments were used to confirm the mutant phenotypes.

### Transformation procedure

Protoplasts were prepared as described in [36], with the following modifications. Petri dishes containing 25 ml of a solid PDA medium covered with a cellophane disk were inoculated with 5×10^6 ^spores and incubated for 20 hours at 26°C. Mycelia were collected (0.2 g/10 ml) in a lysis buffer (1.2 M MgSO_4_, 10 mM sodium phosphate, pH 5.8, 10 mg/ml Glucanex (Novozymes, Bagsvaerd, Denmark), 10 mg/ml mutanase (Interspex Products, San Mateo, CA, USA), and incubated for 2–3 hours at 26°C with gentle agitation. Protoplasts were separated from cell debris by addition of 5 ml of ST buffer (0.8 M Sorbitol, 100 mM Tris-HCl pH = 7.5) and centrifugation at 2000 g for 10 min. The protoplasts collected at the interface of the two solutions were harvested by centrifugation at 750 ***g ***for 5 min, washed twice in ice-cold buffers, once in ST and then in STC (0.8 M Sorbitol, 50 mM CaCl_2_, 100 mM Tris-HCl pH = 7.5; [36]). They were resuspended in 4/5 STC, 1/5 50% PTC buffer (50% PEG 3350 [Sigma-Aldrich], 100 mM CaCl_2_, 100 mM Tris-HCl, pH 7.5) at a final concentration of 5–10 × 10^7 ^protoplasts/ml and kept on ice.

For transformation, 5 μg or 10 μg of DNA suspended in a maximum of 50 μl TE buffer (10 mM Tris-HCl, pH 7.5, 1 mM EDTA) were mixed gently with 100 μl protoplasts and placed on ice. After 30 min, 900 μl of PTCS buffer (50% PTC containing 0.8 M sorbitol) was added and incubation continued at room temperature for 30 min. Aliquots of the protoplasts were mixed with 2.5 ml molten (37°C) top PDAS (PDA with 0.8 M sucrose and 0.3% w/v agar), and overlaid onto 30 ml of selective medium (PDAS containing 8 μg/ ml of hygromycin B. Plates were incubated at 26°C and colonies appeared within 2–4 days. Numerous small colonies stopped growing after 2–3 days. These were interpreted as abortive transformants. Only the colonies that grew after this delay were considered resistant to hygromycin. When co-transformation experiments were performed, 5 μg of the transforming plasmid was mixed with 5 μg of pBC-Hygro.

### DNA manipulation

All the nucleic acid manipulations were performed using standard methods [37]. For Southern blot analysis, genomic DNA was extracted as described in [38]. The cosmid libraries were made in the pMoCosX vector as recommended by Orbach [39]. The library XSG was obtained with *Xho*I partially digested wt genomic DNA. Determination of gene representation in the XSG library was performed by estimating the number of clones containing conserved genes (*NhNia *encoding the nitrate reductase, *NhHsp70 *and *NhHsp90 *encoding molecular chaperones, *NhTUB1 *encoding β-tubulin, *NhTEF1 *encoding the translation initiation factor eEF1A) by PCR, Southern blot hybridization, or direct cloning using a complementation screen. Each tested gene was represented by 2 to 5 independent clones, indicating that the library contained at least four *N. haematococca *genome equivalents. Library XN was made with *Sal*I partially digested wt genomic DNA following the same protocol as for the XSG library. The sequence of the mutant alleles was performed on two independent PCR amplification products, obtained with oligonucleotides im317 (5'-TGATCAACCTCCACGCACAT-3') and ip2393c (5'-AAGGAGATATCGCGCAGGCT-3'). Identification of the 3' end of the *SesA *and *SesB *genes was done by 3'-RACE using the 5'/3' RACE kit (Roche Diagnostics, Meylan, France) on polyA^+ ^mRNA extracted from 2-day-old mycelium, using for *SesA *oligonucleotide ip238 (5'-AAGAGGCCGTGAGACAAGAG-3'), and for *SesB *ip974c (5'-CCAAGACGACCGATAGAAGA-3').

### GenBank accession

The genBank accession no. for cosmid XN31E6 sequences is AY572411.

## Authors' contributions

SG performed all the experimental work and contributed to the interpretation of the data. PS and MJD performed part of the phylogenetic analysis, contributed to the interpretation of the data, and supervised the work. The writing of the manuscript was done in teamwork. All authors read and approved the final manuscript.

## Figures and Tables

**Table 1:** Mutagenesis efficiency and spectrum of mutants

Mutagen^a^	Total number of irradiated thalli	Total number of fast-growing sectors	Types of mutants^b^		
			
			***nas***	s*	***s***
UV_1_	12	15	15	-	-
UV_2_	26	53	53 (13)	-	-
UV_3_	21	40	39 (6)	1 (s*_11_)	-
UV_4_	28	50	49 (16)	1 (s*_27_)	-
UV_5_	44	75	74	1 (s*_4_)	-
UV_6_	21	17	17	-	-
NG_1_	28	25	22	2	1(s_1_)
NG_2_	22	38	37	1 (s*_18_)	-
NG_3_	26	42	37	5	-
Total	228	354	343	11	1
		1.5 per dish	*97*	*3*	

^a^UV, 254 nm UV irradiation; NG, *N*-methyl-*N*'-nitro-*N*-nitrosoguanidine. ^b^*nas *mutants differentiate neither Anneaux nor Secteurs at 26°C but some may do so at other temperatures. Their number are indicated in brackets; s* mutants differentiate Anneaux but not Secteurs, though they have the σ determinant throughout the mycelium; *s*, cultures differentiate Anneaux but not Secteurs, and never contain the σ determinant.

**Table 2:** Results of crosses involving mutants of the Secteur

Crosses	Progency analyzed	Wild-type progeny	
		
		No.	%
*s *× s*_27_	3256	5	0.15
*s*_1 _× s*_18_	3488	7	0.20
*s*_1 _× s*_789_	3824	9	0.25
*s*_1 _× s*_4_	2217	11	0.50
*s*_1 _× s*_84_	1977	10	0.65
*s*_1 _× s*_11_	1051	7	0.65
s*_789 _× s*_84_	2944	3	0.1
s*_789 _× s*_4_	1095	1	0.1
s*_789 _× s*_11_	1808	2	0.1
s*_789 _× s*_27_	2346	1	0.05
s*_789 _× s*_18_	2139	6	0.3
*s*_1 _× *s*_2_	1657	7	0.40

**Table 3:** Phenotypes of heterokaryotic mycelia from various pairings

Strains paired	Prototrophic mycelium^a^	Conclusion
*arg*_154 _(+) / *lys*_255 _(+)	Gray, AS	
*arg*_131_(s*_789_)/*lys*_255_(s*_789_)	Red, As*	
*arg*_154_(s*_84_)/*lys*_255_(s*_84_)	Red, As*	
*arg*_154_(*s*_1_) / *lys*_255_(_*s*1_)	Gray, As	
*arg*_154_(*s*_1_) / *lys*_255_*(+)*	Gray, AS	s_1 _recessive
*arg*_154_(s*_789_) / *lys*_255_*(+)*	Gray, AS	s*_789 _recessive
*arg*_154_(s*_84_) / *lys*_255_*(+)*	Gray, AS	s*_84 _recessive
*arg*_154_(*s*_1_) / *lys*_255_(s*_789_)	Gray, AS	Complementation
*arg*_154_(*s*_1_) / *lys*_255_(s*_84_)	Gray, AS	Complementation
*arg*_154_(*s*_1_) / *lys*_255_(s*_18_)	Gray, AS	Complementation
*arg*_131_(s*_789_) / *lys *_255_(s*_84_)	Red, As*	No complementation
*arg*_154_(s*_18_) / *lys*_255_(s*_84_)	Red, As*	No complementation

^a^AS differentiates both Anneaux and Secteurs and synthesize α and σ only in the respective modified areas. As* differentiates Anneaux but not Secteurs, though they have the σ determinant throughout the mycelium; As differentiates Anneaux but not Secteurs and never contain the σ determinant.

**Table 4:** *Nectria haematococca *strains used

		Characteristics^b^			
			
Strain	Origin^a^	Mycelium	Fertility	Differentiation	Source
Wild type	CBS225/58	Gray	FFF	AS	
s*_789_	NG	Red	F	As*	Daboussi-Bareyre et al. 1979
s*_27_	UV	Red	F	As*	This study
s*_4_	UV	Red	F	As*	This study
s*_11_	UV	Red	F	As*	This study
s*_18_	UV	Red	F	As*	This study
s*_84_	sp	Red	F	As*	Parisot et al. 1981
s_1_	NG	Gray	FFF	As	This study
s_2_	IM	Gray	FF	As	This study
61	UV	Gray	F	AS with a high frequency of Secteur	Daboussi 1985
a_1_	Sp	Gray	FFF	aS	Daboussi-Bareyre et al. 1979
a*_58_	NG	Red	F	a*S	Daboussi-Bareyre et al. 1979
arg_154_	NG	Beige, Smooth arginine-auxotroph	F	AS	Daboussi-Bareyre 1980
lys_255_	NG	Beige, Smooth lysine-auxotroph	F	AS	Daboussi-Bareyre 1980
I_4_mod	cross	Gray	St, white and empty perithecia	AS	Babai-Ahary et al. 1982

^a^CB: Centraal Bureau voor Schimmelcultures, Baarn, The Netherlands. sp, spontaneous mutation; NG, UV and IM recovered after *N*-methyl-*N*'-nitro-*N*-nitrosoguanidine, ultraviolet irradiadiation (254 nm) or insertional mutagenesis, respectively. cross: constructed by crossing. ^b^Main phenotypic characteristics. FFF, very fertile; F, fertile; St, sterile. AS differentiates both Anneaux and Secteurs and synthesizes α and σ only in the respective modified areas. As* differentiates Anneaux but not Secteurs, though they have the σ determinant throughout the mycelium; As differentiates Anneaux but not Secteurs and never contain the σ determinant; a*S differentiates Secteurs but not Anneaux, though they have the α determinant throughout the mycelium; aS differentiate Secteurs but not the Anneaux and never contain the α determinant.
